# The evolution of hearing and balance

**DOI:** 10.7554/eLife.44567

**Published:** 2019-02-08

**Authors:** Forrest P Weghorst, Karina S Cramer

**Affiliations:** 1 Department of Neurobiology and BehaviorUniversity of California, IrvineIrvineUnited States; 2 Neurobiology and BehaviorUniversity of California, IrvineIrvineUnited States

**Keywords:** auditory, vestibular, hindbrain, development, evolution, fate mapping, Chicken, Mouse

## Abstract

New genetic tools have allowed researchers to compare how the brainstem auditory and vestibular nuclei develop in embryonic chicks and mice.

**Related research article** Lipovsek M, Wingate RJ. 2018. Conserved and divergent development of brainstem vestibular and auditory nuclei. *eLife* **7**:e40232. doi: 10.7554/eLife.40232

The ear is an organ with two main roles – hearing and balance – and it relies on mechanically sensitive hair cells to perform both jobs. When the ear detects a sound, it sends signals to clusters of neurons called the brainstem auditory nuclei, and when it registers movement of the head, it sends signals to the brainstem vestibular nuclei. The vestibular nuclei are remarkably similar among vertebrates, from fish to humans (Fritzsch et al., 2014). However, the auditory nuclei display considerable diversity across species. For example, birds and mammals both have a pathway that uses the difference in the time of arrival of a sound at each ear to determine where the noise came from. However, this circuit works differently in these two groups of animals, suggesting that it may have emerged independently multiple times during evolution (Grothe and Pecka, 2014). How, then, did the modern auditory and vestibular nuclei arise?

One strategy to address this question is to explore the embryonic development of vertebrates. Nuclei in the brainstem arise from the embryonic hindbrain, which is remarkably similar across vertebrate species and is divided into segments called rhombomeres (Di Bonito and Studer, 2017). Fate mapping studies have been used to test whether cells in the auditory and vestibular nuclei of different vertebrate species derive from the same rhombomeres.

In this technique, embryonic tissue can be labeled with an external marker, such as a dye (for traditional fate mapping), or a fluorescent protein marker (for genetic fate mapping), in order to track the destination of the cells arising from that tissue (Stern and Fraser, 2001; Legué and Joyner, 2010). Previously, traditional fate mapping has been limited to research in birds (which have accessible embryos), while genetic fate mapping has been limited to mammals (which have accessible genomes; Cramer et al., 2000; Marín and Puelles, 1995; Kim and Dymecki, 2009).

However, both approaches have technical caveats and they identify progenitors in different ways, which hinders their direct comparison. Traditional fate mapping identifies the fate of all labeled cells within a restricted area of the embryo, which may contain a range of progenitor cell types. In contrast, genetic fate mapping marks only cells that express a certain gene (or genes), but these cells can come from a wider area within the embryo.

Now, in eLife, Marcela Lipovsek and Richard Wingate of King’s College London report how they have addressed this dilemma by using vector-based genetic fate mapping in chick embryos (Lipovsek and Wingate, 2018). The researchers focused on genes that were only active in certain regions of the embryonic hindbrain. Plasmid vectors were used to label cells with a fluorescent protein after specific genes in those cells were active. This way, the fate of cells along two anatomical axes (from head to tail, and from back to belly) could be traced. Lipovsek and Wingate studied the same genes that were previously used to construct genetic fate maps of brainstem nuclei in mice, which enabled them to draw direct comparisons between birds and mammals for the first time (Di Bonito and Studer, 2017).

Their results confirmed that vestibular nuclei have similar embryonic origins in chicks and mice. In contrast, auditory nuclei that have comparable roles in chicks and mice arise from completely different embryonic tissues. This suggests that birds and mammals used different populations of ancestral cells – often from different rhombomeres – to independently evolve circuits for calculating sound location.

Lipovsek and Wingate provide compelling evidence that the anatomical similarities between vestibular nuclei in birds and mammals are due to common developmental and evolutionary origins. And since their vestibular systems are also homologous to those of fish, it seems that the role of the vestibular organ remained relatively unaffected by our aquatic ancestors’ move to land (Fritzsch et al., 2014). Conversely, the different developmental origins of auditory nuclei in birds and mammals reflect how each clade solved the problem of hearing on land by adapting to its own ecological niche (Carr and Christensen-Dalsgaard, 2016).
